# Facing ostracism: micro-coding facial expressions in the Cyberball social exclusion paradigm

**DOI:** 10.1186/s40359-023-01219-x

**Published:** 2023-06-19

**Authors:** Rosa H. Mulder, Marian J. Bakermans-Kranenburg, Johan Veenstra, Henning Tiemeier, Marinus H. van IJzendoorn

**Affiliations:** 1 Department of Child and Adolescent Psychiatry/Psychology, Erasmus MC, University Medical Center Rotterdam, Rotterdam, 3000 CB the Netherlands; 2 Generation R Study Group, Erasmus MC, University Medical Center Rotterdam, Rotterdam, the Netherlands; 3 Institute of Education and Child Studies, Leiden University, Leiden, the Netherlands; 4 ISPA - University Institute of Psychological, Social and Life Sciences, Lisbon, Portugal; 5 Department of Social and Behavioral Science, Harvard T.H. Chan School of Public Health, Boston, USA; 6 Department of Psychology, Education and Child Studies, Erasmus University Rotterdam, Rotterdam, the Netherlands; 7 Research Department of Clinical, Education and Health Psychology, Faculty of Brain Sciences, UCL, University of London, London, UK

**Keywords:** Social exclusion, Facial expressions, Observational study, Cyberball, Epidemiology

## Abstract

**Background:**

Social exclusion is often measured with the Cyberball paradigm, a computerized ball-tossing game. Most Cyberball studies, however, used self-report questionnaires, leaving the data vulnerable to reporter bias, and associations with individual characteristics have been inconsistent.

**Methods:**

In this large-scale observational study, we video-recorded 4,813 10-year-old children during Cyberball and developed a real-time micro-coding method measuring facial expressions of anger, sadness and contempt, in a multi-ethnic population-based sample. We estimated associations between facial expressions and self-reported negative feelings, explored associations of child characteristics such as sex and parental national origin with observed and self-reported feelings during social exclusion, and tested associations of observed and self-reported feelings during social exclusion with behavior problems at age 14.

**Results:**

Facial expressions of sadness and anger were associated with self-reported negative feelings *during* the game, but not with such feelings *after* the game. Further, girls reported to have had less negative feelings during the game than boys, but no such sex-differences were found in total observed emotions. Likewise, children with parents of Moroccan origin reported less negative feelings during the game than Dutch children, but their facial expressions did not indicate that they were differently affected. Last, observed emotions related negatively to later internalizing problems, whereas self-report on negative feelings during the game related positively to later internalizing and externalizing problems.

**Conclusions:**

We show that facial expressions are associated with self-reported negative feelings during social exclusion, discuss that reporter-bias might be minimized using facial expressions, and find divergent associations of observed facial expressions and self-reported negative feelings with later internalizing problems.

**Supplementary Information:**

The online version contains supplementary material available at 10.1186/s40359-023-01219-x.

## Background

Humans are a highly social species. As cooperation has been a necessary tool for survival throughout evolution [1, 2], social interaction is an integral part of human behavior. Not surprisingly, humans are remarkably reactive to exclusion from such interaction [3]. Measuring reactions to social exclusion can help to gain insight in the workings of this basic human system for social exclusion reactivity, as well as gain insight into how inter-individual variation in reactions to social exclusion relate to socio-emotional functioning.

Reactions to ostracism, i.e. social exclusion or being ignored, [4] are often studied in the lab with the Cyberball game. In this computerized ball-tossing game, the participant is excluded from the game by two or more avatars [5, 6]. The paradigm has been shown to consistently induce an experience of social exclusion, even when participants are explicitly told that the avatars are not real, that the course of the game is preconceived [7], when receiving the ball is tied to a monetary penalty [8], or when participants are led to believe they are excluded by members of a detested organization such as the Ku Klux Klan [9]. Across the board, Cyberball induces negative emotions and threatens fundamental needs of self-esteem, control, belonging, and meaningful existence [5, 6]. Due to its simplicity and effectiveness, Cyberball has proven to be a valuable tool in understanding behavioral and affective responses to social exclusion in humans.

### Individual differences in responses to social exclusion

Individual differences in the reactions to Cyberball have been associated with differences in personal characteristics. For example, self-esteem was lower [10] and basic needs were more threatened after social exclusion for younger than for older children [11]. Also, females had lower self-esteem [12] and felt more ignored and excluded in reaction to social exclusion [13]. Associations between reactions to Cyberball and individual characteristics, however, are not consistently detected. For example, other studies did not find age [14, 15] or gender differences [10]. In his review on ostracism, Williams mentions that the effects of ostracism seem very resilient to moderation by individual factors such as age, sex, and country of origin [4]. In a meta-analysis of 120 Cyberball studies, a medium-sized moderation effect of personal or situational variables was found for the impact of social exclusion in Cyberball, but a considerable funnel-plot asymmetry suggested substantial publication bias [5].

Several possible reasons for the inconsistent results of Cyberball studies with regards to their associations with individual characteristics have been identified. First of all, it might simply be that the Cyberball paradigm is a victim to its own potency, that is, its strong aversive effect may drown out any associations between individual differences and the reaction to social exclusion. For example, in the study where participants were led to believe that they were excluded by the KKK, those excluded by this despised outgroup reported aversive effect sizes as large as those excluded by a rival outgroup, and as those excluded by in-group members [9]. Hence, these strong overall effects may result in a ceiling effect and/or leave a narrow range of inter-individual variability to associate with individual characteristics. Second, and related to this issue, the research field of Cyberball and individual differences has been plagued by small sample sizes [16, 17], which implies that many studies may not have had the statistical power to detect associations between reactions to social exclusion and differences in personality or psychopathology. Third, many studies [18–20] used self-report questionnaires to measure the reaction to Cyberball as well as to assess individual characteristics. On the one hand, some individuals may not be able to recognize or verbally express their emotions, which could lead to attenuated associations between reactions to Cyberball and individual characteristics. For example, men tend to be less accurate than women to identify and express their own emotions [21]. On the other hand, spurious associations might occur reflecting response biases congruent between self-reported predictor and outcome; individuals with extreme responses on one questionnaire might also be extreme on another, whereas others may have a social desirability bias that masks negative emotions or questionnaire responses, thereby inducing shared method variance bias. A last reason for non-robust results regarding inter-individual differences in reaction to Cyberball might be that the moment of assessment varies between studies [5]. It has been posited that there are two stages relevant to the Cyberball paradigm; 1) the reflexive stage, during which the immediate emotional reaction occurs as a reflex-like response, and 2) the reflective stage, which is thought to be subject to coping mechanisms [5, 22]. For example, it was shown that social anxiety was not related to self-reported negative feelings immediately after the Cyberball, but that socially anxious participants did report more negative feelings 45 min after the game [23]. Yet, recovery from Cyberball may typically set in much faster than that. In a study where participants continuously reported their affect, recovery appeared already during social exclusion and seemed to return to baseline within minutes [24]. Hence different emotion regulation and cognitive coping processes might set in at different stages and relate differently to individual characteristics.

Together, this implies that when measuring associations between personal characteristics and reactions to Cyberball, one would preferentially use not only self-report data after social exclusion, but also observational data during the task to assess the response to the Cyberball paradigm.

### Observation of facial expressions

Observed facial expressions are a prime candidate to gauge immediate emotional reactions. The facial expression system is automatic and involuntary, as well as universal as per its evolutionary basis reflected in the expressions of our primate relatives [25, 26], and is active without the presence of onlookers [27]. Together, this implies that facial emotional expressions may be more resilient to social desirability bias than self-report.

To measure emotional facial expressions, several methodologies exist. Facial electromyography (EMG) allows for the measurement of facial muscles such as the corrugator supercilii (involved in frowning), the zygomaticus mayor (involved in smiling), and the orbicularis oculi (involved in Duchenne smiling) [28]. Using EMG in Cyberball, it has been found that participants showed more orbicularis oculi activation during inclusion than exclusion, presumably utilizing smiling as an affiliative function [29]. In another study using EMG, corrugator supercilii activity increased over time during exclusion versus inclusion, which was taken as indication that social exclusion increased negative affect [30]. As such, EMG is a valuable tool to measure changes in positive and negative emotionality, yet does not optimally allow for distinction between different emotions, as for example activity of the corrugator supercilii is expected in both facial expressions of anger and of sadness [31]. And importantly, the intrusive nature of the method may increase arousal and might reduce possibilities for a naturalistic set-up [32]. Second, a more recent development is that of automated facial expression coding from video material [33]. However, similar to EMG measurement, thus far these techniques cannot be applied to a more naturalistic setting where participants move their upper bodies and head freely, and the field is still working on techniques to mitigate bias in multi-ethnic samples [34]. More traditional human-coded methods, such as the EMFACS (Friesen & Ekman: EMFACS-7: emotional facial action coding system, unpublished), which is based on the coding of discrete action units representing different muscle groups [31], have the drawback that they treat emotions as entirely separate entities, whereas blends both in the facial expression as well in the experience of emotion occur often [35]. Second, these methods are time consuming, with coding time for half an hour for a minute long video [36, 37]. Given that one needs a large sample size to be able to detect small effects, this presents a practical obstacle. A coding methodology that allows for the fast coding of pure and blended emotions is therefore needed.

### Reaction to social exclusion as a time-varying process

When measuring reactions *during* social exclusion, one may tap into different processes than when measuring them shortly *after*. Emotion suppression, concealing outward displays of emotions as they occur (or keeping a ‘poker-face’) [38, 39], is a regulatory mechanism that has been found to be positively associated to depression and internalizing behavior problems [40, 41], while not associated [42] or negatively associated [41, 43] to aggression and externalizing behavior problems. This may mean that facial expressions during social exclusion would be negatively associated with internalizing problems, but positively with externalizing problems. In contrast to facial expressions, as children reporting more threatened needs have been found to have more internalizing problems [18], self-reported negative feelings may be *positively* associated with internalizing problems. No research has been reported on externalizing problems and self-report in Cyberball, but we carefully speculate that as for internalizing problems, these are positively associated.

### The current study

In this large-scale observational study, we video-recorded the faces of nearly 5,000 10-year old children while they played the Cyberball game and developed a new real-time micro-coding method to measure expressions of anger, sadness and contempt during social exclusion. We investigate the utility of these measures by studying associations between observed emotions and conventional self-reported feelings in relation to social exclusion. Further, we explore associations of child characteristics such as sex, age, and parental national origin with observed emotions and self-reported feelings. Lastly, we test associations of observed emotions and self-reported feelings in reactions to social exclusion and parent-reported internalizing and externalizing behavior problems at 14. We hypothesize that the observed facial expressions and self-reported responses to social exclusion have shared variance, yet distinct associations with child characteristics – since earlier research found inconsistent associations of individual differences and response to social exclusion, we have no hypothesis regarding the direction of these associations. Finally, we hypothesize that whereas self-reported negative feelings relates positively to both internalizing and externalizing behavior problems at a later age, observed facial expressions relate negatively to internalizing, and positively to externalizing problems.

## Methods

### Study design

The study took place in the population-based cohort of the Generation R Study [44]. Children came to the research center with their primary caretaker at the age of 10 and played the Cyberball social exclusion paradigm as part of a larger testing procedure, including interviews and physiological measurements. Facial expressions of sadness, anger, and contempt during the game were recorded and micro-coded. Children filled out a post-Cyberball questionnaire regarding their feelings during and after the game. In a series of analyses, i) the observed facial expressions were associated with self-reported feelings, ii) both facial expressions and self-report on feelings during the game were associated with child sex, age, non-verbal IQ, ethnicity, and maternal education, and iii) facial expressions and self-report on feelings during the game were associated with parent-reported child internalizing and externalizing behavior problems at age 14.

### Setting

This study is part of the Generation R Study, a prospective population-based birth cohort that follows children and their parents from pregnancy onwards. Pregnant women residing in the municipality of Rotterdam, the Netherlands, with an expected delivery date between April 2002 and January 2006 were invited to participate in the study. An extensive report on the design of the study can be found elsewhere [44]. Here, we report all measures, manipulations and exclusions relevant to the current analyses. The Generation R Study is conducted in accordance with the World Medical Association Declaration of Helsinki and has been approved by the Medical Ethics Committee of the Erasmus Medical Center, Rotterdam. Written informed consent was obtained from all parents and children over 12 years old, informed assent was obtained from all children younger than 12.

### Study population

In the Generation R Study, 9,778 pregnant mothers had 9,749 live-born children. At the age of 10 years, 5,862 of these children visited the research center and 5,708 children performed the Cyberball task. The data of 5,214 children was usable; 586 had procedural issues (*n* = 22, e.g. the child did not understand the game, or was eating while playing the game), technical issues (*n* = 202, e.g. the game was not working, or the video recording was not working), or were excluded because the child was not visible for more than 30 s (*n* = 362). Of these children, 4,813 filled out the final version of the questionnaire as well (Supplementary Fig. 1). The data of these 4,813 children were used for the main analyses. For the secondary analyses, the data of children were used that had complete data on parent-reported behavior problems at the age of 14 years, resulting in a sample of 3,546 children.

Attrition analyses (Supplementary Table 1) indicated that children in the initial sample (*n* = 9,749) on average had more older siblings (SMD = 0.09, *p* < 0.05), their mothers less often had completed university (SMD = 0.05, *p* < 0.05), and less often had parents that were both born in the Netherlands (SMD = 0.14, *p* < 0.05) than the children in the final sample (*n* = 4,813). The selection effects are in line with earlier reports of lower follow-up rates among children with lower socio-economic status [44]. Further, children who visited the research center at 10 years (*n* = 5,862) did not differ from the final sample for the current paper (*n* = 4,813) on these characteristics, but were on average somewhat older than the children in the final sample (SMD = 0.05, *p* < 0.05). Lastly, analyses indicated that children with data on parent-reported behavior problems at age 14 (*n* = 3,546), were on average slightly younger when visiting the center at age 10 (SMD = 0.11, *p* < 0.05), their mothers more often had completed university (SMD = 0.08, *p* < 0.05), and their parents were more often born in the Netherlands (SMD = 0.14, *p* < 0.05).

### Procedure

Cyberball was administered during the lab visit at the age of 9.8 (SD = 0.3) years. The child sat behind a computer and was told that they would play a ball-tossing game with two other children and asked to imagine the game was happening in real life [45]. The Cyberball program started with a screen that gave the impression that the Cyberball game was played online with two other players. Once the game started, the child saw two pictures of the two putative fellow players, which were children of the same sex as the participating child. The participating child was represented with a baseball glove and when they received the ball they could chose to which picture they tossed the ball by pressing one of two arrow keys. The game consisted of 42 continuous ball tosses. The Inclusion Period consisted of the first six tosses, during which the child received the ball twice (toss 3 and toss 6) and lasted on average 11.6 s (SD = 6.0). The Exclusion Period seamlessly followed upon the Inclusion Period and consisted of 36 tosses which lasted on average 52.4 (SD = 2.8) seconds. During the Exclusion Period, the child received the ball only twice (toss 15 and toss 25), which was done to retain the child’s attention. Unbeknownst to the child, the webcam was turned on to allow recording of the facial expressions. After another, unrelated, dexterity task lasting two minutes, the child was asked to fill out the post-social exclusion questionnaire on their emotions on the computer. During both the Cyberball game and the questionnaire, the experimenter was absent. After completion of the questionnaire, the child was debriefed.

### Facial expressions

**Fig. 1:**
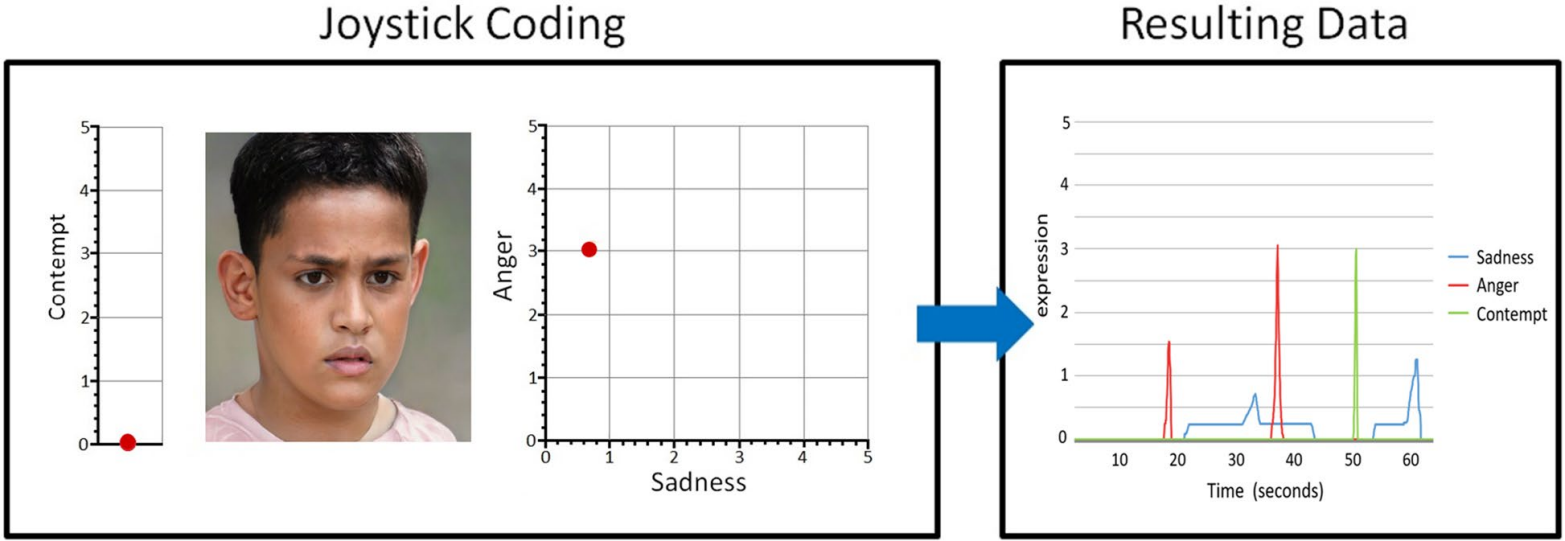
Visual representation of scoring system and resulting data. Image of child obtained from generated.photos

From the webcam videos, the three most often facially expressed emotions; sadness, anger, and contempt, were micro-coded by four coders. We developed a method to micro-code the facial expressions in real-time. To this end, a Java program was developed [46] that picked up the motions of a joystick and throttle (Thrustmaster Inc) and represented them in two graphs alongside each video, which allowed the scoring of three emotions at the same time: sadness, anger, and contempt (code freely available at https://github.com/AVeenstra/JavaJoyMon). Similar methods have successfully been applied to other observational settings, in which dyadic interpersonal behaviors were coded [47]. With the joystick, a dot could be moved along an x- and y-axis in one graph, with the axes representing sadness and anger, respectively. Movement of the throttle affected the movement of a dot along a single y-axis in a second graph, representing contempt. This system allowed for the micro-coding of multiple emotions at the same time (Fig. 1).

The micro-coding method for the emotions was adapted from Ekman’s coding of discrete muscle movements [48]. Sadness was coded if the corners of the mouth were dragged downwards and/or the inner corners of the eyebrows upwards and inwards. Anger was coded if the eyebrows moved inwards and downwards and/or the lips were tightened. Contempt was coded if one of the corners of the mouth was pulled outwards. The intensity and combination of movements influenced the height of the score (0–5), which was continually scored by the position of the joystick and throttle and allowed for the computation of an area under the curve (AUC), representing both the time and intensity of the emotion.

To determine inter-rater reliability, a subset of 129 videos was scored by all four coders. Intercoder reliability (ICC, single measure, absolute agreement) was fair for sadness (ICC = 0.44), and good for anger (ICC = 0.62), contempt (ICC = 0.70) and total negative emotion (ICC = 0.68) [49].

For the current study, area under the curve (AUC) was computed for the Inclusion and Exclusion Period for each emotion (*r* = 0.15–0.22 Inclusion and Exclusion Period). Data of children were only included if the child’s face was visible for at least 8 s during the Inclusion Period and at least 30 s during the Exclusion Period. Each AUC was corrected for the total amount of time the child was visible and represents the average AUC per 10 s. The Exclusion Period score was adjusted for baseline facial expressions in the shorter Inclusion Period by residualizing the Exclusion Period score on the Inclusion Period score. The facial expression scores thus reflect both the total time and intensity of emotion during the Exclusion Period, adjusted for emotional expressions during the Inclusion Period. A total negative emotion AUC was computed by summing the AUCs of sadness, anger, and contempt. In case of uncertainty in the coding, a second opinion was asked from another coder (2% of videos). Since residualized AUC data for the individual emotions had a positive skew, the residualized AUCs for sadness, anger and total negative emotion were logarithmically scaled (Supplementary Fig. 2). Since the contempt AUCs had a clear bimodal distribution, with the largest peak with the lower scores of children who did not show any contempt, the score for contempt was dichotomized.

### Post-social exclusion questionnaire

The post-social exclusion questionnaire included 15 questions and was adapted from questionnaires used in previous Cyberball studies [6, 7, 50]. The questions were presented as written statements and through audio via headphones. The first nine questions concerned the feelings of the child at the moment of reporting, i.e. after the game; the six subsequent questions concerned the emotions of the child during the game. Questions concerned mood (e.g. ‘I feel happy’; ‘I was happy during the ball game on the computer’) and self-esteem (e.g. ‘My self-esteem is high’; ‘My self-esteem was high during the ball game on the computer’). The questions regarding the feelings of the child during the game also included items on feelings of control (e.g. ‘The others decided everything during the ball game on the computer’) and belonging (‘I felt like I belonged to the group during the ball game on the computer’), and the questions regarding the feelings of the child after the game also covered meaningful existence (e.g. ‘I feel invisible’). Each item was scored on a Likert-scale from 1 (‘Not at all’) to 5 (‘Very much’).

To examine the dimensional structure of the post-social exclusion questionnaire an exploratory factor analysis was performed, which groups the individual items by their covariance structure (Supplementary Table 2). Exploratory factor analysis was performed with the *nFactor* R package [51] in R version 3.6.1 [52]. Two items; ‘I feel important’ and ‘I feel invisible’ were excluded from the analyses due to low factor loadings (< 0.40) in the various factor solutions and because we suspected that these figurative forms of statement were not suitably translated for this Dutch 9-year-old population. A scree plot of the post-social exclusion questionnaire items showed that a three-factor solution was the most optimal, and an exploratory factor analysis with orthogonal rotations (‘varimax’) led to three factors. However, since the second and the third factor tapped into similar constructs and the third factor included only three items, we decided for a two-factor solution, which also showed a good fit (RMSEA = 0.086 and TLI = 0.82). The first factor included six items regarding the child’s feelings during the game (Eigenvalue = 3.6, 19% explained variance), the second factor included seven items regarding the child’s feelings after the game (Eigenvalue = 2.2, 16% explained variance). Positively phrased items were reversed, and the subscales were labelled ‘Negative feelings during the game*’* and *‘*Negative feelings after the game*’*, respectively (Supplementary Fig. 3)*.* Reliability of *‘*Negative feelings during the game*’* was good with a Cronbach’s alpha of 0.81, and that of *‘*Negative feelings after the game*’* was acceptable with a Cronbach’s alpha of 0.72.

### Child characteristics

Child sex and date of birth were obtained from midwife- and hospital registries. Information on maternal education and parental national origin was obtained via a questionnaire at enrollment. Maternal education was used as a proxy of socio-economic status (SES) and dichotomized into children whose mothers had completed a university study (scored as 1) and those who had not (scored as 0). Parental national origin was based on the parent’s country of birth [53]. The mother’s country of birth was used in cases where both parents were born abroad. Non-verbal intelligence (IQ) of the child was measured with the Snijders-Oomen Non-verbal intelligence test – Revised (SON-R 2.5–7) [54] at the mean (SD) age of 6.1 (0.4) years. The IQ-score was based on the scores from two subsets with each 15 items; ‘Mosaics’ for visuospatial abilities, and ‘Categories’ for abstract reasoning.

### Behavior problems

Child behavior problems were measured via the Child Behavior Checklist/6–18 (CBCL) [55], as reported by the primary caretaker (mother in 95% of cases) at the mean (SD) age of 13.5 (0.4) years. Items were rated on a 3-point scale (0 = ‘not true’;1 = ’somewhat or sometimes true’; 2 = ’very true or often true’), regarding problem behavior in the past 6 months. We used the broadband scales Internalizing Problems (covering syndrome scales Anxious/Depressed, Withdrawn/Depressed, and Somatic Complaints with 32 items) and Externalizing Problems (covering Rule-breaking Behavior and Aggressive Behavior with 35 items). For each of the CBCL total scores, 25% missingness on the items was allowed, sum scores were weighted accordingly. The CBCL has been shown to have good validity and reliability [55] and to be generalizable across 23 societies, including the Dutch [56, 57]. In the current sample, the internal consistency was α = 0.87 for Internalizing Problems and α = 0.88 for Externalizing Problems.

### Statistical analyses

All analyses were performed in R version 3.6.1 [52]. Missing data on child characteristics and predictors of interest (*n* = 397 for education of the mother, *n* = 712 for non-verbal IQ, *n* = 114 for parental national origin) were imputed using the *mice* R package [58] using a maximum of 100 iterations creating 30 datasets. All results are based on pooled estimates of the multiply imputed sets.

Basic characteristics of the facial expressions were studied on the group level in three ways: (1) by examining the time-course of the facial expressions by computing a 3-s moving average, (2) by comparing the AUC of the Inclusion and Exclusion Period, averaged over the duration of each respective period, for each child separately by computing the difference between the two AUCs and on a group level by paired t-tests of the two logarithmically scaled AUCs, and (3) by studying the maximum intensity of each micro-coded facial expression by computing the median highest peak for each child.

Associations between observed facial expressions (exposure: total negative emotion, sadness, anger, or contempt) and self-reported feelings in the post-social exclusion questionnaire (outcome: ‘Negative feelings during the game’ or ‘Negative feelings after the game’) were estimated in linear regressions. Each combination of facial expressions and post-social exclusion questionnaire scale was adjusted for by child characteristics (child sex, age, maternal education, non-verbal IQ, and parental national origin). To explore influences of reporter bias, associations between both observed emotion (facial expressions of total negative emotion) and reported feelings (Negative feelings during the game) with child characteristics were estimated using linear regressions with the child characteristics as predictors and either observed emotions or reported feelings as the outcome. To study associations of reactions to Cyberball (facial expressions of total negative emotion and Negative feelings during the game) and later behavior problems, linear regressions were performed with observed emotions or reported feelings as predictor and parent-reported internalizing or externalizing problems at age 14 as the outcome, adjusted for child characteristics. Last, in a series of sensitivity analyses, analyses were repeated using robust linear regressions to test results for robustness against residual non-normality of our measures and linear regressions were repeated within the non-imputed dataset (*n* = 4,033 or *n* = 3,093 for analyses of behavior problems).

Since novel measurements were tested in this study, we did not correct for multiple testing and set α at 0.05. Together with the sample size of *N* = 4,813, this allowed us (two-tailed significance criterion, statistical power = 0.80, number of predictors = 6) to detect small effect sizes of f^2^ = 0.002 and above. For *N* = 3,546 (behavior problems), this value was similarly f^2^ = 0.002.

## Results

**Fig. 2:**
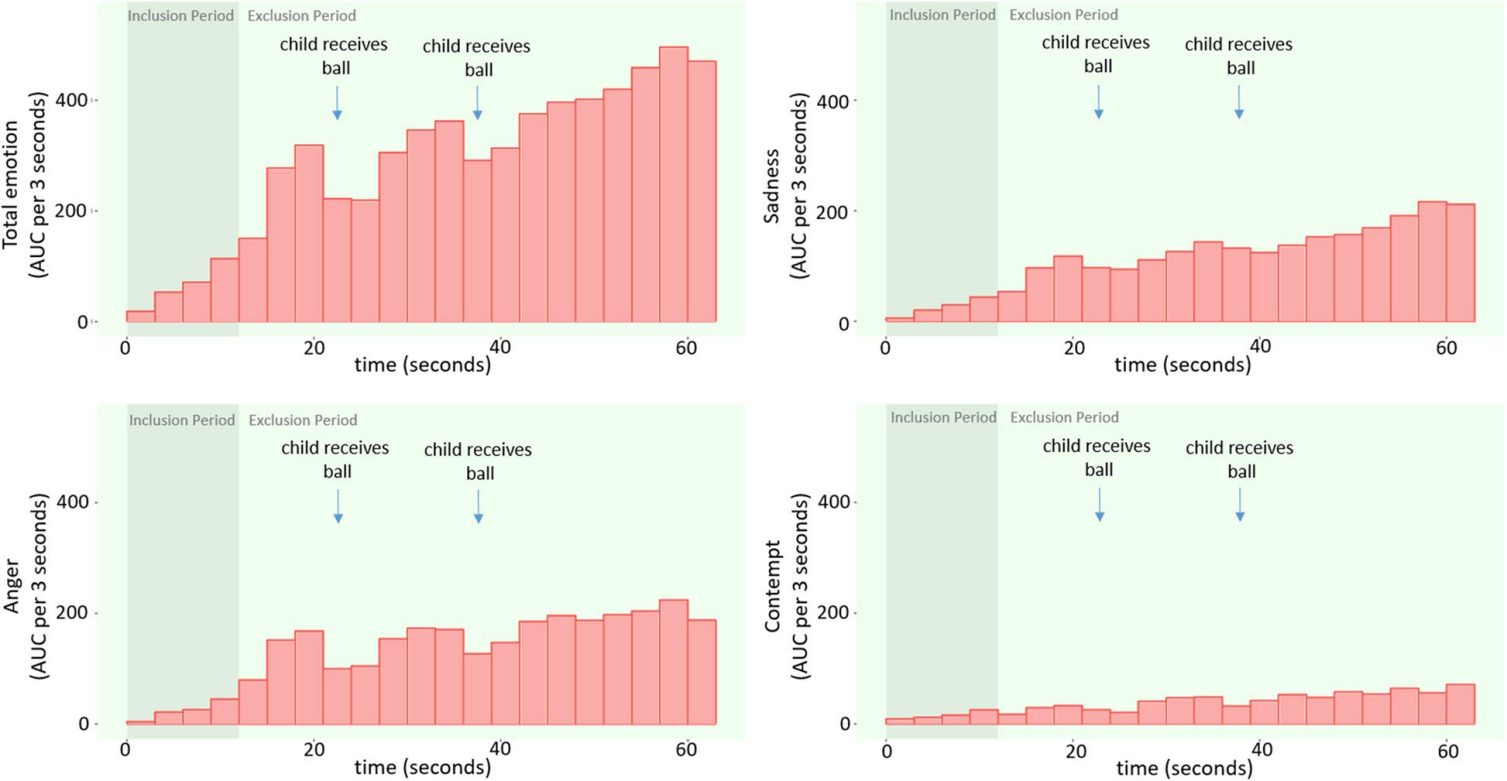
Mean area under the curve (AUC) over each three seconds of the Cyberball time course

Sample characteristics are displayed in Table 1. The time-course of the facial expressions was studied on a group level by computing a 3-s moving average. Figure 2 shows that the group average expression increased over the course of the Cyberball paradigm but that the expression was less intense each time the child received the ball. This pattern was present for each negative emotion coded. Most children had negative facial expressions during the paradigm: 75.1% showed sadness, 70.1% showed anger, 27.1% showed contempt and 98.4% showed either of those three emotions. A comparison of the AUC score for the Inclusion Period and the Exclusion Period showed that most children had more negative facial expressions during the Exclusion Period than during the Inclusion Period. Out of the children that showed that respective emotion during the paradigm, 75.0% showed more sadness, 70.8% showed more anger, 24.0% showed more contempt, and 87.9% showed more total negative emotion during the Exclusion Period than during the Inclusion Period. Paired t-tests indeed showed that Exclusion AUC was higher than Inclusion AUCs on a group-level for all emotions: sadness (t[4812] = 58.82, *p* < 2.2e-16), anger, (t[4812] = 55.28, *p* < 2.2e-16), contempt (t[4812] = 21.20, *p* < 2.2e-16), and total negative emotion (t[4812] = 73.40, *p* < 2.2e-16).

**Table 1:** Sample characteristics

Sex, No. girls (%)	2445 (50.8)
Age at Cyberball in years, mean (SD)	9.80 (0.34)
Education of the mother, No. university (%)	1282 (26.6)
Non-verbal IQ, mean (SD)	101.70 (14.97)
Parental national origin, No. (%)	
Dutch	2856 (59.3)
non-Dutch, Western	433 (9.0)
non-Dutch, non-Western	1524 (31.7)
Facial expressions – Total negative emotion, mean AUC/10 s, mean (SD)	13.35 (17.29)
Facial expressions – Total negative emotion, *log transformed* mean AUC/10 s, mean (SD)	1.46 (0.18)
Facial expressions – Sadness, mean AUC/10 s, mean (SD)	5.28 (8.88)
Facial expressions – Sadness, *log transformed* mean AUC/10 s, mean (SD)	1.56 (0.15)
Facial expressions – Anger, mean AUC/10 s, mean (SD)	6.32 (11.94)
Facial expressions – Anger, *log transformed* mean AUC/10 s, mean (SD)	1.24 (0.20)
Facial expressions – Contempt, mean AUC/10 s, mean (SD)	6.32 (11.94)
Facial expressions – Contempt, any contempt No. yes (%)	1228 (25.5)
Questionnaire – Negative feelings during the game, mean (SD)	2.85 (0.84)
Questionnaire – Negative feelings after the game, mean (SD)	1.57 (0.45)
Internalizing problem score, mean (SD)	5.5 (5.7)
Externalizing problem score, mean (SD)	4.1 (5.1)

The correlations between the AUCs for the different facial expressions are depicted in Table 2. Sadness and anger were positively associated (*r* = 0.13, *p* = 2.07 × 10^–19^), as were anger and contempt (*r* = 0.03, *p* = 0.042). No association was found between sadness and contempt. The two scales of the post-social exclusion questionnaire, ‘Negative feelings during the game’ and ‘Negative feelings after the game’ correlated positively (*r* = 0.26, *p* < 2.40 × 10^–73^).

**Table 2:** Correlations between facial expressions during social exclusion

	Sadness	Anger	Contempt
Total negative emotion	0.63**	0.75**	0.21**
Sadness		0.13**	-0.01
Anger			0.03*

^*^ *p* < 0.05

^**^ *p* < 0.0001

### Observed emotions and self-reported feelings

Results of linear regressions between the AUCs of the facial expressions and self-reported feelings in the post-social exclusion questionnaire showed that observed total negative emotion, sadness, and anger associated positively with ‘Negative feelings during the game’ (β = 0.09, β = 0.09, and β = 0.06, respectively, *p* ≤ 1.06 × 10^–05^) (Table 3). Contempt was not associated with ‘Negative feelings during the game’. There were no associations between the facial expressions and ‘Negative feelings after the game’. Since observed total negative emotion was associated with self-reported feelings during the game, but not after the game, we contrasted only observed total negative emotion with self-reported feelings during the game with respect to their associations with child characteristics.

**Table 3:** Associations between observed emotions during social exclusion and reported feelings in post-social exclusion questionnaire

*Self-report*	Negative feelings during the game			Negative feelings after the game		
	β	95% CI	*p*	β	95% CI	*p*
*Observed facial expression*						
Total negative emotion	0.09	0.06; 0.12	1.04 × 10^–10^	0.00	-0.03; 0.02	0.77
Sadness	0.09	0.06; 0.12	4.94 × 10^–10^	0.00	-0.03; 0.03	0.93
Anger	0.06	0.04; 0.09	1.06 × 10^–05^	0.00	-0.03; 0.03	0.84
Contempt	0.01	-0.06; 0.07	0.81	-0.01	-0.07; 0.06	0.77

Analyses were adjusted for sex, age, maternal education, non-verbal IQ, and parental national origin

### Child characteristics in relation to observed emotions and reported feelings

Associations between child characteristics and the individual observed emotions with good inter-rater reliability, i.e. anger and contempt, were estimated (Supplementary Table 3). Boys showed less anger than girls did, and less anger was observed in children with parents of Dutch national origin than in children with parents of non-Dutch Western or non-Dutch non-Western national origin. No associations were found between anger and age of the child, maternal education, or non-verbal IQ. Older children showed less contempt. No other associations with contempt were found.

Associations between child characteristics and observed negative emotion as well as reported negative feelings are presented in Table 4. Sex, age, maternal education, child IQ, and parental national origin were not associated with observed negative emotion. Several associations were found between child characteristics and self-reported negative feelings during the game. Girls reported less negative feelings during the game (β = -0.17, *p* = 3.40 × 10^–09^) than boys did, as did children of mothers who completed university versus those whose mothers did not (β = 0.08, *p* = 0.026), and children with higher versus lower non-verbal IQ (β = 0.07, *p* = 7.48 × 10^–05^). Children of parents with non-Western national origin reported less negative feelings during the game (β = -0.14, *p* = 2.76 × 10^–05^).

**Table 4:** Associations between child characteristics and observed emotions and reported feelings during social exclusion

	Observed total negative emotion			Self-reported negative feelings during the game		
	β	95% CI	*p*	β	95% CI	*p*
Sex (girl)	0.03	-0.03; 0.08	0.34	-0.17	-0.23; -0.12	3.40 × 10^–09^
Age	0.01	-0.02; 0.04	0.44	0.00	-0.03; 0.03	0.84
Maternal education (high)	-0.01	-0.08; 0.06	0.75	0.08	0.01; 0.15	0.03
Non-verbal IQ	0.02	-0.01; 0.06	0.19	0.07	0.04; 0.11	7.48 × 10^–05^
Ethnicity – non-Dutch Western^a^	0.09	-0.02; 0.19	0.10	0.02	-0.08; 0.12	0.74
Ethnicity – non-Western^a^	0.06	-0.01; 0.13	0.07	-0.14	-0.21; -0.08	2.76 × 10^–05^

Analyses for each predictor were mutually adjusted the other predictors

^a^Reference group: Dutch

Last, since non-Western children reported strikingly lower levels of negative feelings during the game, we performed a follow-up analysis on the specific ethnic groups within our non-imputed sample (*n* = 4,033), for all reactions to social exclusion (Supplementary Table 4). Results showed that children of Turkish or Moroccan parents reported fewer negative feelings during the game and following this up one step further, children of Moroccan parents in particular reported fewer negative feelings after the game (Supplementary Table 5), but these children did not show less negative facial expressions during the game. 

### Observed emotions and self-reported feelings in relation to later behavior problems

No associations were found between observed anger or contempt and later internalizing or externalizing behavior problems (Supplementary Table 6). The associations between observed total negative emotion as well as self-reported negative feelings during the game with behavior problems 4 years later are shown in Table 5. Children who displayed less total negative emotion during Cyberball had more internalizing problems 4 years later, whereas no association with externalizing problems was found. On the other hand, children who reported *more* negative feelings during Cyberball had more internalizing as well as externalizing problems at age 14.

**Table 5:** Reaction to social exclusion at 10 years and parent-reported behavior problems at 14

	Internalizing problems			Externalizing problems		
	β	95% CI	*p*	β	95% CI	*p*
Observed total negative emotion	-0.04	-0.07; -0.00	0.03	-0.02	-0.05; 0.02	0.36
Self-reported negative feelings during the game	0.07	0.04; 0.11	9.18 × 10^–06^	0.06	0.03; 0.10	2.23 × 10^–04^

Analyses were adjusted for sex, age, maternal education, non-verbal IQ, and parental national origin

### Sensitivity analyses

First, analyses were repeated using robust linear regressions. Results for associations between facial expressions and post-social exclusion questionnaire scales were confirmed using these analyses (Supplementary Table 7). Results for associations between child characteristics and facial expressions were similarly consistent (Supplementary Table 8), and results for associations between reactions to Cyberball and later behavior problems were as well (Supplementary Table 9). Second, analyses were repeated on a set without imputation of child characteristics (*n* = 4,033 in main analyses and *n* = 3,093 in secondary analyses on behavior problems). Results for associations between facial expressions and post-social exclusion questionnaire scales were confirmed in this set (Supplementary Table 10). Results for associations between child characteristics and facial expressions were in line with results for the imputed data (Supplementary Table 11), as were the results for associations between reactions to Cyberball and later behavior problems (Supplementary Table 12).

## Discussion

In the current study, we aimed to introduce and examine a system for the fast micro-coding of facial expressions in the Cyberball social exclusion paradigm, a frequently used computerized paradigm simulating social exclusion. In addition, we explored observed emotions and self-reported feelings in association with child characteristics, and studied associations of observed emotions and self-reported feelings with later behavior problems. In a population-based sample of 4,813 10-year-old children, we found that most children showed negative emotional responses to the paradigm as observed from their facial expressions. The observed facial expressions of sadness and anger were associated with post-social exclusion self-report on negative feelings *during* the game, but not with negative feelings *after* the game. The facial expression of contempt was not associated with self-reported emotions during or after the game. In addition, we found that girls, children with a lower IQ, and children with parents of Moroccan national origin, reported less negative feelings during the game, but did not show less (or more) negative emotions than other children as based on their observed facial expressions. Last, in a subsample of 3,546 children, we found that children with negative facial expressions had less internalizing behavior problems four years later, whereas children reporting more negative feelings during the game had more internalizing and externalizing behavior problems four years later.

No association between facial expressions of contempt and self-reported feelings was found. The association between expressions of contempt and anger was not very strong, in line with the literature [59]. In their series of studies, contempt was shown to be related to anger as both often co-occur in an attempt to reassert social status in a negative social interaction. Contempt co-occurred with interpersonal distancing, whereas anger was shown to be a more short-term emotion and associated with reconciliation. Anger may be more adaptive in a social situation as the one simulated in the Cyberball paradigm, which may be why older children showed less contempt than younger children did, but research on contempt during development is scarce, making the interpretation of this finding speculative, and pointing to the need of developmental research on (the expression of) contempt.

### Reactions to social exclusion and individual differences

Exploratory analyses showed that girls displayed more anger than boys did during social exclusion. This finding is in contrast with other research, in which 8-year old boys displayed more anger during social rejection than girls [60]. A developmental perspective might explain the difference between studies; a meta-analytic review revealed that while boys express more anger and girls more sadness during middle childhood, during early adolescence girls show more anger than boys [61]. Another explanation might be that in this age range girls have a stronger preference for equity in social situations than boys [62]. The Cyberball paradigm violates this expectancy, which might frustrate girls even more than boys.

The underreporting of negative feelings by girls, children with a lower IQ, and children with parents of Moroccan national origin may be due to a social desirability bias. In a questionnaire designed to detect social desirability bias, it was shown that girls were more prone to provide socially desirable answers than boys were [63]. In men with and without intellectual disabilities, Langdon, Clare, and Murphy [64] showed that verbal and performance IQ were negatively associated with social desirability bias. Children of immigrant Moroccan parents showed the same amount of negative facial expressions as other children, but they reported to have fewer negative feelings during and after the game. It is estimated that 4% of children in the Netherlands have a first or second generation Moroccan background, but in urban areas such as the city of Rotterdam, that number is much higher [65]. It has been previously shown that children of parents with Moroccan national origin reported to express less negative emotions towards their family members than children from Dutch origin [66], a finding that is concordant with the current observations. Together, these findings underline the importance of observational measures in research to counter reporter biases such as social desirability bias. Furthermore, these findings are relevant for teachers and physicians, indicating that girls, children with a lower IQ, and children with certain cultural backgrounds might not be as vocal about their problems and needs as other children are.

### Reactions to social exclusion and later behavior problems

We found that observed facial expressions during the task and post-Cyberball self-report on feelings during the task had divergent associations with parent-reported internalizing problems four years later. This difference may occur because of the qualitative difference between the two measures, however, timing may have also played a role. Emotion suppression is a regulatory process that is expected to happen at the moment of the stressful interaction itself and it is therefore possible that the observation of facial expressions during social exclusion taps into this process. Given that both emotion suppression and negative self-reported feelings after social exclusion have been related to more internalizing problems [18, 40, 41], this could explain the opposite directionality of the association for observed facial expressions and self-reported feelings. Although more research on emotion regulation strategies is necessary to understand the exact regulatory processes that underlie these divergent associations, our results indicate that the two measures have differential associations with later internalizing behavior problems.

### Strengths, limitations, and future directions

Results showed that observed anger, sadness as well as total negative emotion were associated with self-reported negative feelings during the game, but given that the presently used questionnaire was targeted at a large epidemiological sample which limited testing time and thus included a modest number of items, we were unable to verify that observed anger specifically associated with self-reported anger, and observed sadness with sadness. In future research, a questionnaire would be needed that measures each emotion as a separate entity. In addition, EMG measurement of facial muscles would have allowed to verify the intensity of the observed facial expressions, however, the naturalistic set-up of the paradigm in did not allow for this measurement. Further, time constraints of this epidemiological study did not permit a true experimental design with a full inclusion as well as exclusion condition. By adjusting the score for emotions observed during the brief inclusion period, however, we controlled for baseline facial expressions not related to social exclusion. Due to the non-random order of the inclusion and exclusion phase, we also could not determine if the increase in facial expressions of negative emotions during the exclusion versus inclusion phase was due to the social exclusion, or because emotions increased over time. However, we note that intensity of emotions seemed to specifically decrease during the two times that the participant received the ball in the exclusion phase, suggesting that there is a relation between exclusion and observed negative emotions.

One of the goals of the study was to study emotional facial expressions in a naturalistic setting, allowing for the observation of blended emotions. Whilst the inter-rater reliability of anger and contempt where good, that of sadness was suboptimal. This might be attributed to the fact that sadness and anger were often presented in concert, which has also been observed by others [35].

The size of the current population-based sample allowed for the detection of small effect sizes. Associations found between facial expressions and self-report were highly significant, which decreases the chance that these were false positives (yet does not insulate results from inherent biases). Most of the reported associations detected in the explorative analyses between child characteristics and reactions to social exclusion were also highly significant, but the association of maternal education would not survive a multiple testing correction and warrants replication. Overall, the large majority of the children in the current study reacted with facial expressions of negative emotions. This observation is in line with an earlier study on social exclusion and facial expression [67] and with results from Williams [4] and Hartgerink et al. [5], who noted that the Cyberball paradigm has pervasive negative effects on participants. Further, the authors observed that effects of Cyberball are relatively robust against moderation by personal or situational factors. Here we found a consistent association of observed emotions with reported feelings, indicating that there is meaningful inter-individual variation in the reaction to Cyberball. Since associations with individual characteristics for self-report were not corroborated by the observations, we suggest researchers measure responses to Cyberball both by self-report and observation.

## Conclusions

This is the first time that this fast micro-coding method was employed to study emotional facial expressions during social exclusion. Results showed concurrent validity with self-reported feelings during the game. Our results also suggest that self-report questionnaires may be subject to a social desirability response bias in specific groups of children, and that facial expressions and self-report are differentially related to internalizing behavior, highlighting the complementary value of observational methods for emotions during challenging social settings.

## Supplementary Information

**Additional file 1: Supplementary Fig. 1.** Flow chart of participant inclusion.

**Additional file 2: Supplementary Fig. 2.** Distribution of log transformed values of facial expressions of total negative emotion, sadness, and anger.

**Additional file 3: Supplementary Fig. 3.** Distribution of scores for self-reportednegative feelings during the game andnegative feelings after the game.

**Additional file 4: Supplementary Table 1.** Attrition analyses.

**Additional file 5: Supplementary Table 2.** Post-social exclusion questionnaire and loadings on exploratory factor analysis.

**Additional file 6: Supplementary Table 3.** Associations between child characteristics and observed facial expressions during social exclusion.

**Additional file 7: Supplementary Table 4.** Differences in reactions to social exclusion for children of different ethnicities.

**Additional file 8: Supplementary Table 5.** Differences in reactions to social exclusion for Turkish and Moroccan children versus Dutch children.

**Additional file 9: Supplementary Table 6.** Associations between observed facial expressions during social exclusion at 10 years and parent-reported behavior problems at age 14.

**Additional file 10: Supplementary Table 7.** Associations between observed emotions during social exclusion and reported feelings in post-social exclusion questionnaire using robust linear regressions.

**Additional file 11: Supplementary Table 8.** Associations between child characteristics and observed emotions and reported feelings during social exclusion using robust linear regressions.

**Additional file 12: Supplementary Table 9.** Reaction to social exclusion at age 10 years and parent-reported behavior problems at age 14 using robust linear regressions.

**Additional file 13: Supplementary Table 10.** Associations between observed emotions during social exclusion and reported feelings in post-social exclusion questionnaire in non-imputed set.

**Additional file 14: Supplementary Table 11.** Associations between child characteristics and observed emotions and reported feelings during social exclusion in non-imputed set.

**Additional file 15: Supplementary Table 12.** Reaction to social exclusion at 10 years and parent-reported behavior problems at age 14 in non-imputed set.

## Data Availability

De-identified data from the Generation R Study are available upon request (generationr@erasmusmc.nl), subject to local rules and regulations. Code for statistical analyses is available upon request (generationr@erasmusmc.nl). Code for the Java program for joystick coding is available online (https://github.com/AVeenstra/JavaJoyMon).

## Abbreviation

AUC: area under the curve
