# Supplementary figures and images for: Facing ostracism: micro-coding facial expressions in the Cyberball social exclusion paradigm
Source: BMC Psychol. 2023 Jun 19;11:185. doi: 10.1186/s40359-023-01219-x (PMC10280895; doi:10.1186/s40359-023-01219-x)

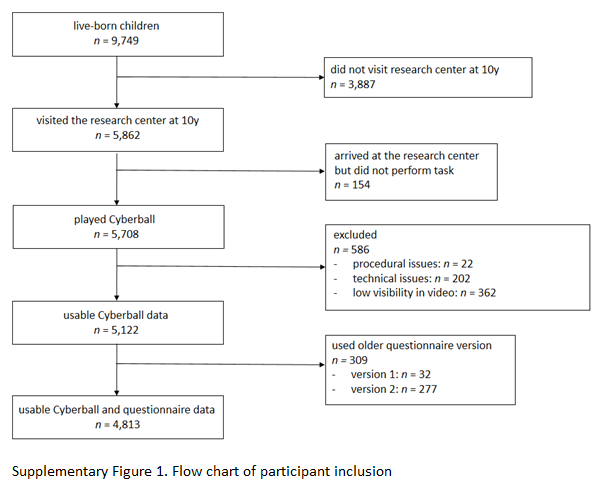

Supplement: Supplementary file 1 — Additional file 1: Supplementary Fig. 1. Flow chart of participant inclusion. [file 40359_2023_1219_MOESM1_ESM.png]

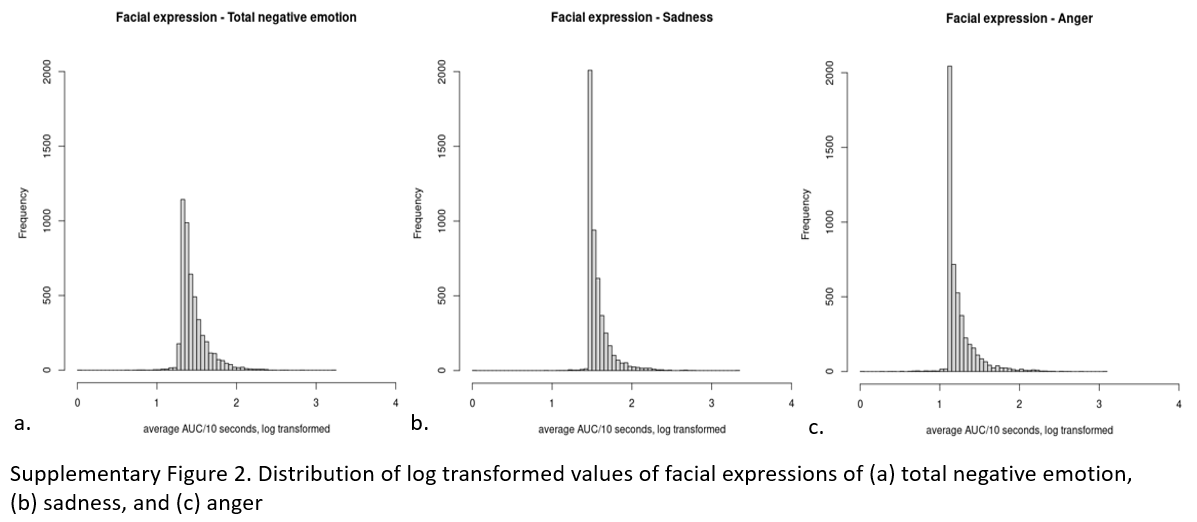

Supplement: Supplementary file 2 — Additional file 2: Supplementary Fig. 2. Distribution of log transformed values of facial expressions of total negative emotion, sadness, and anger. [file 40359_2023_1219_MOESM2_ESM.png]

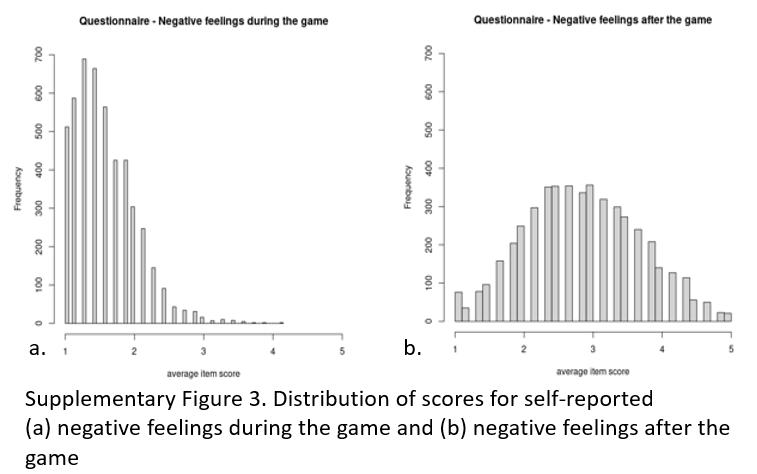

Supplement: Supplementary file 3 — Additional file 3: Supplementary Fig. 3. Distribution of scores for self-reportednegative feelings during the game andnegative feelings after the game. [file 40359_2023_1219_MOESM3_ESM.png]
